# A comparison of intimate partner violence and associated physical injuries between cohabitating and married women: a 5-year medical chart review

**DOI:** 10.1186/s12889-016-3879-y

**Published:** 2016-11-29

**Authors:** Janet Yuen-Ha Wong, Anna Wai-Man Choi, Daniel Yee-Tak Fong, Edmond Pui Hang Choi, John Kit-Shing Wong, Fung Ling So, Chu-Leung Lau, Chak-Wah Kam

**Affiliations:** 1 School of Nursing, Li Ka Shing Faculty of Medicine, The University of Hong Kong, 4/F, William M.W. Mong Block, 21 Sassoon Road, Pokfulam, Hong Kong SAR; 2 Department of Social Work and Social Administration, Faculty of Social Science, Room 534, Jockey Club Tower, The Centennial Campus, The University of Hong Kong, Pokfulam, Hong Kong SAR; 3 Department of Accident and Emergency Medicine, Tuen Mun Hospital, Tuen Mun, Hong Kong SAR; 4 Department of Accident and Emergency Medicine, Pok Oi Hospital, Yuen Long, Hong Kong SAR

**Keywords:** Intimate partner violence, Physical violence, Injury, Cohabitation, Women

## Abstract

**Background:**

Cohabitation, referring to a co-residential romantic relationship between two intimate partners without a marriage license, has become widely accepted in contemporary societies. It has been found that cohabitating women have a higher risk of experiencing intimate partner violence (IPV) than married women. However, as yet, no studies have investigated the level and pattern of IPV-associated physical injuries and its mental health impact on cohabitating women. Therefore, we aim to compare IPV-associated physical injuries between cohabitating and married women by conducting a review of 5-year medical records from the emergency departments of two major public hospitals in Hong Kong.

**Methods:**

This is a retrospective cohort study. Using two computerized systems, we identified the medical charts of 1011 women who had experienced IPV and presented at emergency departments between 2010 and 2014, of which, 132 were cohabitating and 833 were married.

**Results:**

Cohabitating women were significantly younger (*p*-value < .0001) and had obtained a higher educational level (*p*-value = .008) than married women. After adjusting for those two variables, the logistic regression models showed that cohabitating women were approximately 2.1 times more likely than married women to present with head, neck, or facial injuries (OR = 2.1, 95% CI = 1.30–3.40, *p* = .002), and the risk of having multiple injuries in different locations (head, neck, face, torso, limbs) was almost twice that for cohabitating women compared with married women (OR = 1.82, 95% CI = 1.25–2.65, *p* = .001). Furthermore, cohabitating women were almost two times as likely as married women to experience more than one method of physical violence (OR = 1.72, 95% CI = 1.18–2.51, *p* = .005). There were no significant differences regarding mental health, police reporting, and discharge plans.

**Conclusions:**

Owing to recent social changes to the family structure, including the growing acceptance of cohabitation, it is essential that a screening program for IPV is established for cohabitating women, as well as the inclusion of IPV content in medical and nursing curriculums and in-service training.

## Background

Intimate partner violence (IPV), whether in a cohabitating or married relationship, can occur in the form of physical and sexual abuse, stalking, and psychological aggression (including coercive tactics) by a current or former intimate partner [1]. IPV is a public health issue that impacts on both the healthcare system and the economy. The US National Violence Against Women Survey and the Medical Expenditure Panel Survey have reported that abused women experience more physical and mental injuries, higher healthcare utilization, and a higher loss of productivity in paid work than non-abused women [2]. Furthermore, physically abused women may suffer from different types of injuries, including head and neck injuries and musculoskeletal injuries [3]. A US study of 3333 women reported that physically abused women had more visits to emergency and hospital outpatient departments, and to primary care, pharmacy, and specialty services than non-abused women [4]. Consequently, the annual health care costs were 42% higher for physically abused women. These representative data from both governmental reports and community studies imply that IPV-associated injuries should be further investigated to develop effective primary interventions.

Cohabitation, referred as a co-residential intimate relationship without an official marriage license, is becoming more and more acceptable in contemporary society [5]. The Centers for Disease Control and Prevention National Health Statistics Reports of Family Growth interviewed 12,279 women aged 15–44 and reported an increase of cohabitation rates for women, increasing from 34% in 1995 to 48% in 2006–2010 [6]. This rising rate may be due to women obtaining higher levels of both education and economic status in the 20^th^ century [7].

Although the majority of cohabiting relationships are pre-marital, post-marital cohabitation with the former spouse after divorce has also been reported [8]. Cohabitating can provide co-residential intimacy and a family-like environment with a more egalitarian family structures and a low level of economic consolidation [9]. However, cohabitation can be seen to deliver a weakened relationship bond without an inherent barrier against separation [10]. Hence, cohabitating partners might not have any barrier to dissolution if IPV occurs. Consequently, one may expect less IPV in cohabiting relationships than in married relationships [11]. Empirical evidence has demonstrated the contrary however, that physical violence in cohabitating relationships is at least twice as common as in married relationships [11, 12]. Furthermore, these studies about the physical violence in cohabitating relationships were based on the study samples in 1980s [11, 12]. The study findings might no longer be applicable. Little update-to date information is available about couples that cohabitated or married. Considering the substantial increase in the prevalence of cohabitations recently, the association between physical violence and cohabitating relationship should be re-examined. While considerable attention has been paid to IPV suffered by married women, little is known about IPV experienced by women in cohabitating relationship. Specifically, no study has yet investigated the level of IPV-associated physical injury and its mental health impact on women in cohabitating relationships.

Hong Kong residents view cohabitation similarly to those in the West. Acceptance of cohabitation in Hong Kong increased from 36% in 1981 to 51% in 2008, as reported in a local study of 1014 individuals [13]. Despite Hong Kong society being based on Chinese norms and traditions, its history as a British colony has provided Hong Kong with a mixed culture, with an openness to Western values and beliefs, especially demonstrated in younger generations. In fact, a local study found that younger respondents were more likely to accept cohabitation than older respondents [13]. However, cohabitating women may not be supported by their parents, leading to less available resources if IPV occurs. Therefore, Hong Kong is a relevant place to investigate the context of IPV-associated physical injury experienced by cohabiting women.

Numerous studies examining the IPV-associated physical injuries of abused women have been conducted in emergency rooms to take advantage of the routine classification of the types and causes of injuries [14]. Again, no study has specifically focused on cohabitating women. Therefore, the present study aims to compare IPV-associated physical injuries between cohabitating women and married women in the context of their location and frequency, type of physical violence, mental impact, as well as the relationship problems initiating the conflict and violence, willingness to report to police, and discharge plans. We hypothesize that physical violence and injuries are more severe in cohabitating women than in married women suffering from IPV [15, 16]. The results may indicate the need to obtain comprehensive information to develop a screening program for IPV victims including cohabitating women.

## Methods

### Study design

This is a medical record review of all IPV-associated physical injuries experienced by women presenting at the emergency department of two local public hospitals in Hong Kong between 2010 and 2014. The hospitals serve catchment areas with the highest annual incidence of IPV in Hong Kong. In addition, IPV-associated injuries and marital status were directly assessed by the triage nurses in both hospitals. The nurses were trained for assessing IPV and used direct questioning to the women when suspicious was present. Ethical approval for this study was obtained from the Ethics Committee of the Hong Kong Hospital Authority, which governs all public hospitals in Hong Kong. Following approval, the Medical Records Offices granted the permission for the collection of medical records for this study.

The steps of this review process are as follows: (1) data abstraction instrument development; (2) medical records retrieval; (3) data abstraction; and (4) data checking. As the present study aims to compare abused cohabitating and married women, only the medical charts with indication of IPV during consultation at the emergency departments and their data were included and presented.

1. Data abstraction instrument development
  A data abstraction instrument was developed for data collection from medical charts. The first two authors identified the study variables, which included the date and time of admission and injuries associated with physical violence, details of injury site, type of physical violence, frequency of abuse, and whether a police report was made. We also collected the following data: woman’s age, educational level, employment status, residency in Hong Kong, marital status, relationship problems with the perpetrator and mental impact, and discharge destination. The procedures and treatments carried out at the emergency department were also recorded. However, the procedures and treatments were beyond the scope of the present study and were not reported. The instrument was developed in such a way that all variable information could be obtained from the medical, nursing, psychology, and social worker reports or hospital records, which were particularly designed for IPV.
  Coding was designed and marked on the data abstraction instrument. For each variable, coding was consolidated into categorical or dichotomous options to facilitate efficient data collection. The instrument was independently trialed on 20 medical charts by the first two authors. Quality checking was conducted after the completion of 20 medical charts. Discrepancies were resolved in a consensus meeting. The eighth author, who is a physician, was consulted to resolve any issues regarding illegible handwriting. Upon consensus, the instrument was revised with the addition of some missing coding to avoid ambiguity and was then transferred to an electronic Excel template for data abstraction. The electronic template was installed on several laptop computers and further tested on five medical charts to ensure that all the data were obtained in an Excel format after data entry.
2. Medical record retrieval
  All medical records between 2010 and 2014 were first retrieved from two computerized systems: the Accident and Emergency Information System (AEIS) and Clinical Data Analysis & Reporting System (CDARS) in public hospitals in Hong Kong. AEIS holds medical records from the emergency departments while CDARS keeps records of hospitalized in-patients and out-patients. CDARS was used because some women with IPV may not be identified at the emergency department but during hospitalization. The fifth and eighth authors accessed and identified all IPV records from the two computerized systems, based on which the corresponding medical charts indicating domestic violence were retrieved from the Medical Records Office. The information can be located from a bubble optical answer on the medical charts, which indicated whether the client had spousal abuse, child abuse, elder abuse, sibling abuse, indecent assault or no abuse during the consultation at emergency department. All retrieved medical charts were then manually checked to confirm they concerned women who had experienced IPV.
  A total of 1647 medical charts were retrieved, of which 1212 recorded IPV; the others recorded child abuse, elder abuse, sibling abuse, indecent assault or no abuse. Among the 1212 medical charts on IPV, 1011 (83.4%) of the victims were women with 132 cohabitating and 833 married women. The remaining medical charts were excluded from the present study because the women were ‘dating’ without cohabitation (*n* = 14), or separated/divorced (*n* = 32).
3. Data abstraction
  To ensure the quality of the data abstraction, the data collectors were nurses with at least 3 years’ work experience in an emergency department and were familiar with the medical records system and writing styles. Before data collection, the nurses were told the study objectives (but were blinded to the study hypothesis), trained to use the electronic data abstract template, and explained the operational definition of each data field. During training, the data collectors were invited to provide feedback on the electronic data abstraction template based on which further modifications and customizations of the electronic template were made. During the data collection process, our study team nurses and clinicians were available to resolve any ambiguities (some “remarks” data fields were added for text input to facilitate such resolution). All data collection was conducted in a secure onsite location to avoid the loss of medical charts and confidential information. Moreover, the data were entered without patient names to ensure anonymity.
4. Data checking
  To assess the reliability of data collection, the first author independently extracted data from a randomly sample of 100 (approximately 10%) medical charts. As missing and incomplete data can be a concern in retrospective medical record reviews [17], the focus was on those fields with missing data during the data checking. The reliability of the data collection method was confirmed at 95% reliability.

### Data analysis

Descriptive statistics were computed to summarize the characteristics of the abused women. A comparison was then made between the characteristics of cohabitating and married women. Using dichotomous independent variables, stepwise logistic regression models were used to examine the context of IPV-associated physical injuries in cohabitating women via a comparison with married women. The following independent variable were studied: head, neck, and face injuries, frequency of physical violence, type of physical violence, relationship problems initiating conflict and violence, impact on women, willingness to report to police, and discharge plans. Odds ratios were also reported with married women as the reference group. Moreover, to minimize the selection effect of cohabitation and marriage, the model results were adjusted by age, education level, and local residency status in Hong Kong because all these variables were found to be significantly different between cohabitating and married women. The goodness of fit of all logistic regression models was assessed using Hosmer and Lemeshow tests [15]. Data analysis was undertaken using IBM SPSS Statistics 23.0 software package (IBM Corp, Armonk, NY, USA). All statistical tests were two-sided and used a 5% level of significance.

## Results

**Fig. 1:**
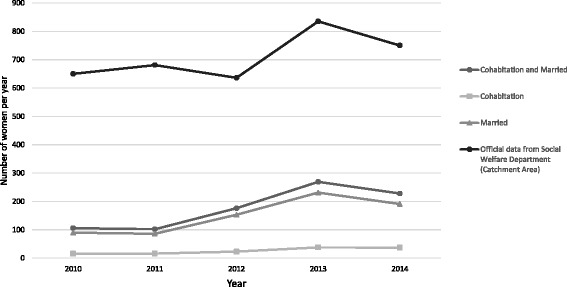
Number of abused women presenting to the emergency departments in Hong Kong

The annual number of abused women presenting at emergency departments between 2010 and 2014 are presented in Fig. 1. The figure shows a moderate increase in the number of abused women. Among the 132 cohabitating women, 128 (97%) were in pre-marital cohabitation while 4 were in a post-marital reunion after divorce. Table 1 summarizes the demographic characteristics of cohabitating and married women. The mean age of cohabitating women was significantly younger than that of married women (married women mean = 39.81, SD = 11.56; cohabitating women mean = 31.29, SD = 9.30, *p*-value < .0001). Almost half of the cohabitating women had obtained upper secondary qualifications or above, which was also significantly higher than married women (*p*-value = .008). The majority of cohabitating women had residency in Hong Kong (*n* = 82, 78.1%), which was significantly higher compared with married women (*p*-value = .03). However, there was no difference in employment status between the two groups.

**Table 1:** Comparison of cohabiting and married women’s characteristics (*n* = 903)

	Number of cases (percentage^a^)/Mean ± SD		
Characteristics	Married women (*n* = 833)	Cohabitating women (*n* = 132)	*p*-value*
Age	39.81 ± 11.56	32.29 ± 9.30	<.0001
Level of education			.008
Primary or below	182/621 (29.3%)	17/89 (18.3%)	
Lower secondary (S1-3)	227/621 (36.6%)	30/89 (33.7%)	
Upper secondary (S4-5)	129/621 (20.8%)	24/89 (27%)	
Matriculation (S6-7), Tertiary or above	83/621 (13.4%)	18/89 (20.2%)	
Residency in Hong Kong	464/677 (68.5%)	82/105 (78.1%)	.03
Employed	190/452 (42%)	37/75 (49.3%)	.26

^a^The percentage had only included the valid cases for comparison whereas missing values were not counted

*p-value of Pearson’s R for ordinal or continuous characteristics (age and level of education); *p*-value of Fisher exact test for binary characteristics (residency and employment status)

The results of the logistic models are reported in Table 2. After adjusting for age, educational level, and local residency status, the odds of a cohabitating woman having a head, neck, or facial injury were 2.1 times that of a married woman (95% CI = 1.30–3.40, *p* = .002), while the odds of having multiple injuries in different locations (head, neck, face, torso, or limbs) was 1.8 times more likely for a cohabitating woman (95% CI = 1.25–2.65, *p* = .001). Among the different types of physical violence, slapping (OR = 2.2, 95% CI = 1.39–3.55, *p*-value = .001), arm twisting or hair pulling (OR = 1.9, 95% CI = 1.08–3.25, *p*-value = .025), pushing (OR = 1.7, 95% CI = 1.05–2.66, *p*-value = .032), and slamming against a wall (OR =2.39, 95%CI1.34–4.25, *p*-value = .016) were all significantly more likely for cohabitating women than for married women. Although there were no significant differences between other types of physical violence, cohabitating women were almost two times (OR: 1.63, 95% CI = 1.03–2.59, *p* = .005) more likely to experience more than one type of physical violence than married women. Among the relationship problems that led to conflict and violence, cohabitating women were 2.3 times (OR: 2.28, 95% CI = 1.15–4.5, *p*-value = .013) more likely to be abused by a perpetrator with a drinking problem than married women, and two times more likely to be abused by someone with a diagnosed mental illness than married women (OR: 2.00, 95% CI = 1.01–3.96, *p*-value = .048). There were no significant differences regarding the mental impact on women, police reporting, and discharge plans.

**Table 2:** Comparison between cohabitating and married relationships: characteristics of physical violence, including method, relationship problems, and impact on women

	Married (*n* = 833)	Cohabitation (*n* = 132)		
	Number (%)	Number (%)	Odds ratio^a^	95% CI
Head-neck-face injury	573 (68.8%)	114 (83.2%)	2.10*	1.30–3.40
Multiple injuries in different locations (i.e., head-neck-face, body truck or limbs)	355 (42.6%)	81 (59.1%)	1.82*	1.25–2.65
Multiple frequency of physical violence (i.e., 3 times or above)	429 (63.5%)	64 (56.1%)	1.00	0.99–1.00
Type of Physical Violence				
Use of weapon	142 (17%)	21 (15.3%)	0.97	0.57–1.66
Throw things that could hurt	88 (10.6%)	12 (8.8%)	1.26	0.70–2.27
Slapping	185 (22.2%)	36 (26.3%)	2.22*	1.39–3.55
Punching with fist	503 (60.4%)	84 (61.3%)	1.09	0.70–1.70
Kicking	111 (13.3%)	23 (16.8%)	1.52	0.88–2.64
Choking	57 (6.8%)	16 (11.7%)	1.86	0.89–3.89
Arm twisting or hair pulling	101 (12.1%)	26 (19.0%)	1.87*	1.08–3.25
Pushing	201 (24.1%)	46 (33.6%)	1.70*	1.06–2.73
Slamming against the wall	77 (9.2%)	22 (16.1%)	2.39*	1.34–4.25
Using more than 1 method of above	355 (42.6%)	81 (59.1%)	1.63*	1.03–2.59
Relationship problems initiated conflicts and violence				
Other relationship affairs involved	99 (11.9%)	19 (13.9%)	1.20	0.66–2.18
Financial problem	24 (7.3%)	4 (6.6%)	1.61	0.60–4.30
Abuser drinking problem	50 (12.9%)	17 (27.9%)	2.28*	1.15–4.50
Abuser use of drug problem	35 (8.2%)	6 (8.8%)	1.17	0.47–2.87
Abuser having diagnosed mental illness	46 (11.5%)	13 (20.6%)	2.00*	1.01–3.96
Mental impact on women				
Women having drinking problem	6 (1.6%)	0 (0%)	1.20	0.34–4.18
Women use of drug	7 (1.6%)	1 (1.4%)	1.99	0.39–10.22
Women having diagnosed mental illness	65 (14.4%)	9 (12.3%)	0.57	0.26–1.26
Report to police	507 (60.9%)	89 (65%)	0.89	0.56–1.42
Discharged to shelter^b^	102 (13.0%)	19 (15.1%)	1.60	0.79–3.24
Discharged to own home^b^	423 (52.5%)	60 (45.1%)	0.73	0.48–1.11
Discharged to relative or friend’s home^b^	127 (16%)	28 (21.9%)	1.63	0.95–2.81
Discharged against medical advice^b^	49 (6.3%)	12 (9.6%)	1.13	0.49–2.62

^a^Stepwise logistic regression analysis results after adjustment for age, education level, and local residency status (*p-value < 0.05)

^b^Sixty-one women were included in the analysis because they were initially hospitalized

## Discussion

This is the first study to investigate the elevated level of physical violence and injuries experienced by cohabitating women in comparison with married women. Data were reliably collected over a 5-year period from the emergency departments of two major public hospitals in Hong Kong. We found that cohabitating women suffer more severe physical violence and injuries than married women, with doubled odds of suffering more than one type of physical violence. Moreover, they are more likely to have head, neck, or facial injuries and multiple injuries over different parts of their bodies.

The higher level of physical violence and injury experienced by cohabitating women when compared to married women can be understood from two perspectives. Cohabitation is traditionally viewed as a “trial marriage”. Using the natural selection theory, partners exhibiting low level or no violence may be more likely to enter into marriage, whereas cohabitating women living in abusive relationships may tend to stay in cohabitating relationships [16]. Alternatively, the Power–Control Theory stipulates status incompatibilities in intimate partner relationships might lead to lower marital satisfaction and even IPV [18–20]. In our context, married women may enjoy a shared family income for family expenses, investment, or other joint ventures, whereas cohabitating women may choose to be more financially independent. Consequently, cohabitating women would follow the equality principles, in particular, for power in relationship and wage-earnings [21]. It follows then that if there is any imbalance in power, tension and conflict in the relationship, or jealousy regarding wage-earnings, this could intensify and escalate into severe physical violence and injury. Moreover, it is suggested that social status and access to financial resources will affect the distribution of power and control within intimate partner relationships. Status incompatibility and status reversal (women have a better financial and social status than their husband) might make women susceptible to IPV because some men are highly threaten by their wife who outrank them in economic power and social status [22, 23].

Some characteristics of people who cohabit might increase the risk of IPV. It was suggested that people who chose to cohabit were more likely to grow up with divorced parents, to have a non-traditional attitudes toward marriage and to be non-religious than people without cohabitation [24]. It was possible that these are contributing factors of IPV. For example, it was found that attendance at religious services was associated with lower rates of IPV victimization among males and females and lower rates of IPV perpetration among male [25]. Furthermore, our subjects who chose to cohabitate were younger than the married subjects. Due to life span development, young people are less likely to have positive relationship skills and more likely to use physical aggression to deal with conflicts in intimate relationships [26, 27]. For further studies, other factors such as religion, marital status of parents, and attitude towards marriage should be collected to further explore the linkage between cohabitation and physical violence.

We found that having a partner with drinking problems is a risk factor of IPV. Strong links between alcohol use and violent behaviours have been well documented in previous studies [28, 29]. A study reported that women whose partners with alcohol abuse were 3.6 times more likely than other women to be assaulted by their partners [30]. Given the fact that alcohol abuse is a contributing factor of IPV, intervention designed to reduce alcohol consumption might lead to the decrease in IPV [29]. Furthermore, for people who chronically engage in IPV, it is important to assess their drinking status. If the perpetrator is a heavy drinker, alcohol interventions might have beneficial impacts [29]. It is equally important to assess the violent behaviours of heavy drinkers for the safety of their partners.

It is not surprising to find that having a partner with mental illness is a risk factor of IPV as mental illness and violence behaviours are found to be related [31]. Nonetheless, there are still clinical implications. First, mental health assessment should be conducted for the perpetrators of IPV. Second, the violent behaviours and the history of abusing partners should be assessed in patients with mental illness for promoting the safety of their partners.

We found no significant differences in drinking problems, substance abuse, and mental health among the abused women, whether they were cohabitating or married. The relatively greater freedom of cohabitating women to separate did not appear to have significantly reduced the mental impact of IPV when compared with married women. In addition, even though the results were non-significant, it was found that the cohabitating women were unlikely to report to police about their abuse. Thus, accompanied with the higher level of physical violence experienced by cohabitating women, both their safety and IPV homicide rates deserve further investigation.

The present study has enriched our understandings of the differences in marital status in violence against women, which is an ongoing social issue. The worldwide increase in cohabitation rates implies that views on marital status are undergoing important social changes. Sociology scholars suggest that the stages of cohabitation can be described as a partnership transition [32]. In the first stage, cohabitation was a deviant phenomenon practiced by a small group of people. In the second stage, cohabitation was regarded as a “trial marriage” or a probation period to test couples’ commitment to marriage. In the third stage, cohabitation became a socially acceptable alternative to marriage, and in the fourth stage, cohabitation and marriage became indistinguishable with children being born and reared in partnership between two cohabitants. Hong Kong, a city characterized as having a mixed culture with both Chinese and Western influences, has made a social transition towards the third stage of development. Traditionally, cohabitation was not an acceptable practice in Chinese society, with a 1981 survey reporting that 56% of respondents were against cohabitation [33]. However, in the last decade or so, cohabitation has been viewed more favorably. In 2008, a government survey among 1014 Chinese adults reported that 69% of participants accepted cohabitation between two adults who planned to marry and 45% accepted cohabitation between two adults with no plans to marry [34]. Moreover, 51% accepted a long-term cohabitation relationship without legal marriage, indicating that cohabitation has become increasingly acceptable. Therefore, it is predicted that cohabitation will become more popular in the future. Hence, both researchers and clinicians should pay greater attention to the topic of violence against cohabitating women.

### Limitations

The present study contains several limitations that can be addressed in future studies. First, the credibility of this study as a medical record review lies on the reliability and completeness of the clinical data. The missing values of the variables in this study ranged from 0 to 26%, which were lower than the data used in our prior study [16], mainly because of the revised data abstraction format and the implementation of the electronic data collection method [35]. Second, although we have collected data regarding the family issues behind the abuse and violence and the mental health impact on women, the information contained in the medical records is limited. For example, the duration of cohabitation was not reported in the medical records, making it difficult to factor in the effects of cohabitation duration in the analysis. Furthermore, the length of relationship is not available in this study. We are not able to control for this variable in the analysis. Last, we only focused on abused women visiting an emergency department, who may have more severe injuries than those in the community. Therefore, the findings may not be generalizable to other populations.

### Implications

The elevated level of physical violence and injuries inflicted upon cohabitating women reveals the need for an IPV screening program for this high-risk group of women. The 2013 World Health Organization clinical and policy guidelines have introduced a clinical pathway for IPV [36]. They recommend the assessment of the exposure to IPV when assessing conditions that may be caused or complicated by IPV; for example, symptoms of depression and anxiety, unexplained chronic pain, and repeated health consultations with no clear diagnosis. In particular, the present study shows that healthcare providers at emergency department should be aware of the appropriate ways in which to enquire about IPV with a focus on the association between cohabitation and IPV. In addition, they should be able to respond to women disclosing IPV by providing an appropriate safety assessment, practical care, referrals to suitable treatment services, and tangible resources covering legal, housing, and childcare services. This package of brief, first-line counseling should be provided with a focus on the needs and responses of the women to reduce further IPV and minimize its impact. That said, as the emergency department is a busy setting and healthcare providers may only have limited time to provide counseling, it is recommended that an on-site counselor or social worker be available for immediate first-line care.

Pre-education and in-service training is crucial to reduce IPV. Unfortunately, the current medical and nursing curriculums in Hong Kong do not make appropriate reference to IPV and related issues [37]. Furthermore, healthcare providers lack in-service training to assess and intervene in IPV. Healthcare providers, particularly those working in emergency departments, have significant opportunities to help vulnerable groups break the cycle of violence by assessing, identifying and treating IPV. Hence, education regarding violence prevention, assessment, risks, impact, and treatment is important to enable healthcare providers to obtain the knowledge and skills to provide competent care to IPV survivors. Abused women who are cohabitating may be seen to deserve less attention, as they often have no children and legal marriage obligation. However, the present study shows their unique status with regard to violence and injury severity; hence, cohabitating women do indeed deserve more attention. The matter of equity of care between cohabitating and married women should also be emphasized in IPV education material.

## Conclusions

Cohabitating perpetrators of IPV are more violent than married perpetrators, thus cohabitating women experience a higher level of physical violence victimization and injury than married women. Because of the recent social changes to family structures with cohabitation now being widely social acceptable, it is essential that screening programs for cohabitating women are established and IPV education is introduced into medical and nursing curriculums.

## Abbreviations

AEIS: Accident and emergency information system
CDARS: Clinical data analysis & reporting system
IPV: Intimate partner violence
